# Care partner needs in Parkinson's disease: A systematic review of qualitative and quantitative data

**DOI:** 10.1177/1877718X251344066

**Published:** 2025-05-30

**Authors:** Max Hulshoff, Christine Sun, Elaine Book, Caroline Tanner, Nabila Dahodwala, Brenda Reynolds, Heather Boon, Connie Marras

**Affiliations:** 1 Department of Neurology, Haga ziekenhuis, Den Haag, The Netherlands; 2 Morton and Gloria Shulman Movement Disorders Centre and the Edmond J Safra Program in Parkinson's Research, Toronto Western Hospital, University of Toronto, Toronto, Ontario, Canada; 3 Pacific Parkinson's Research Centre, University of British Columbia, Vancouver, British Columbia, Canada; 4 Department of Neurology, Weill Institute for Neuroscience, University of California – San Francisco, San Francisco, CA, USA; 5 Department of Neurology, Perelman School of Medicine, University of Pennsylvania, Philadelphia, PA, USA; 6 Independent Researcher, Vancouver, Canada; 7 Leslie Dan Faculty of Pharmacy, University of Toronto, Toronto, Ontario, Canada

**Keywords:** Parkinson’s disease, systematic review, caregiver, spouse, needs

## Abstract

**Background:**

Care for persons with Parkinson's disease (PD) is to a great extent carried out by care partners. It is important to understand their needs to ease their burden and help with their important role.

**Objective:**

To present (1) what is known about needs in caregiving for someone with PD from both qualitative and quantitative papers; and (2) to identify research gaps in the existing literature to guide future research.

**Methods:**

A systematic search was conducted, searching PubMed, CINAHL, PsychINFO, and MEDLINE for both qualitative and quantitative studies examining care partner needs in Parkinson's disease published from the start of the databases up to 13 November 2024. The best-fit framework synthesis method was employed for qualitative data extraction and analysis. The Critical Appraisal Skills Programme (CASP) and the Newcastle-Ottawa Scale (NOS) were used for quality assessment of studies.

**Results:**

Forty-eight qualitative studies, ten quantitative studies, and three mixed methods studies met the eligibility criteria. All studies were of observational, cross-sectional design. A total of nine themes (the need for information, the need to be heard, PD healthcare, emotional support, daily living, financial support, skills, care partner physical well-being, and respite care) were identified from qualitative data and all quantitative data could fit this framework. Quantitative data on the frequency of needs and when they arise over the course of PD were scarce. Only one quantitative study made use of a validated measurement instrument to measure care partner needs, the Family Needs Questionnaire.

**Conclusions:**

Care partner needs in PD are wide-ranging. A significant gap identified is the absence of quantitative data to determine the prevalence, timing, and factor contributing to the needs revealed by the qualitative research.

## Introduction

The care of people with Parkinson's disease (PD) is to a great extent carried out by informal care partners (i.e., spouse, family members).^1–3^ Due to the progressive nature of the disease in motor and cognitive domains, more responsibility for care is gradually placed on care partners over time.^1–4^ Their role as care partners may cause psychological, economic, and social burden, which can lead to negative impact on their own physical and emotional health and lower quality of life.^5–7^ Addressing the needs of care partners may improve the carer's quality of life, and may allow them to maintain their caregiving role which is associated with better patient outcomes, or even strengthen the care partners in their caregiving role, thus improving care for the person with PD.^8^ Interventions focused on support and providing information addressing individual care partner needs have shown promising results in other neurodegenerative diseases such as dementia and multiple sclerosis.^9,10^ The objective of this review is to present (1) what is known about needs in caregiving for someone with PD from both qualitative and quantitative papers; and (2) to identify research gaps in the existing literature to guide future research.

## Methods

Methods and results sections are reported based on the Preferred Reporting Items for Systematic Reviews and Meta-Analysis (PRISMA) criteria.^11^

### Literature search

We performed a systematic search through PubMed, CINAHL, PsychINFO, and MEDLINE on needs in caregiving in PD following a search strategy on PubMed used in a prior review.^5^ A preliminary literature scan on PubMed was performed to identify search terms and to set up eligibility criteria. The strategy shown below was used to identify both qualitative studies and quantitative studies from the start of the databases until 13 November 2024:

(parkinson disease [MesH Terms] OR Parkinson* [tiab]) AND (care partners [MesH Terms] OR Spouse [MesH Terms] OR caretaker* [tiab] OR care partner* [tiab] OR caregiv* [tiab] OR partner* [tiab] OR spouse* [tiab] OR care partner* [tiab])

### Inclusion criteria

Qualitative studies: (1) Reporting on care partner needs, either directly, or indirectly allowing needs to be inferred based on reported burdens; and (2) Published in English.

Quantitative studies: (1) Reporting on care partner needs; and (2) Published in English.

### Exclusion criteria

(1) Studies that involved paid care partners; and (2) Review studies, although the latter were scanned to capture additional relevant papers from their reference list.

### Data collection and analysis

The titles and abstracts of all search results were screened against the inclusion and exclusion criteria by two authors (MH, CS). A sample check was carried out by CM to ensure alignment. The full text of articles that potentially met the eligibility criteria were reviewed and any discrepancies regarding a final determination of inclusion were resolved by discussion between authors. Eligible studies were divided into three categories: qualitative, quantitative, and mixed-methods.

The best-fit framework synthesis method was employed for qualitative data extraction and analysis, where secondary thematic synthesis is premised on a primary conceptual framework. This method uses conceptual frameworks or theories to identify elements to address a specific research question.^12^ The primary conceptual framework used to do the initial qualitative coding was derived using the items of the Support Person's Unmet Needs Survey - Short Form (SPUN-SF), a validated questionnaire on care partner needs for persons with cancer, chosen due to its wide coverage of relevant themes.^13^ The SPUN-SF contains items with the following subscales: information needs, planning for the future, work and finance, health care access and continuity, personal needs, and emotional needs. In the course of thematic synthesis, emergent themes from the qualitative studies were captured and integrated into the framework if the data did not map to any of the pre-existing themes. The coding process was performed using QUIRKOS software^14^ and completed independently by MH and CS. The derivation of themes was adjusted through discussions with all authors. Definitions of themes can be found in the Supplemental Material. Quantitative study results were mapped to the themes developed through qualitative data analysis. Quality assessment was performed independently by MH and CS for individual qualitative studies using the Critical Appraisal Skills Programme (CASP) checklist,^15^ and for individual quantitative studies using the Newcastle-Ottawa Scale (NOS) for cohort studies.^16^ Any disagreements were resolved by consensus.

## Results

### Search results and study characteristics

**Figure 1.**
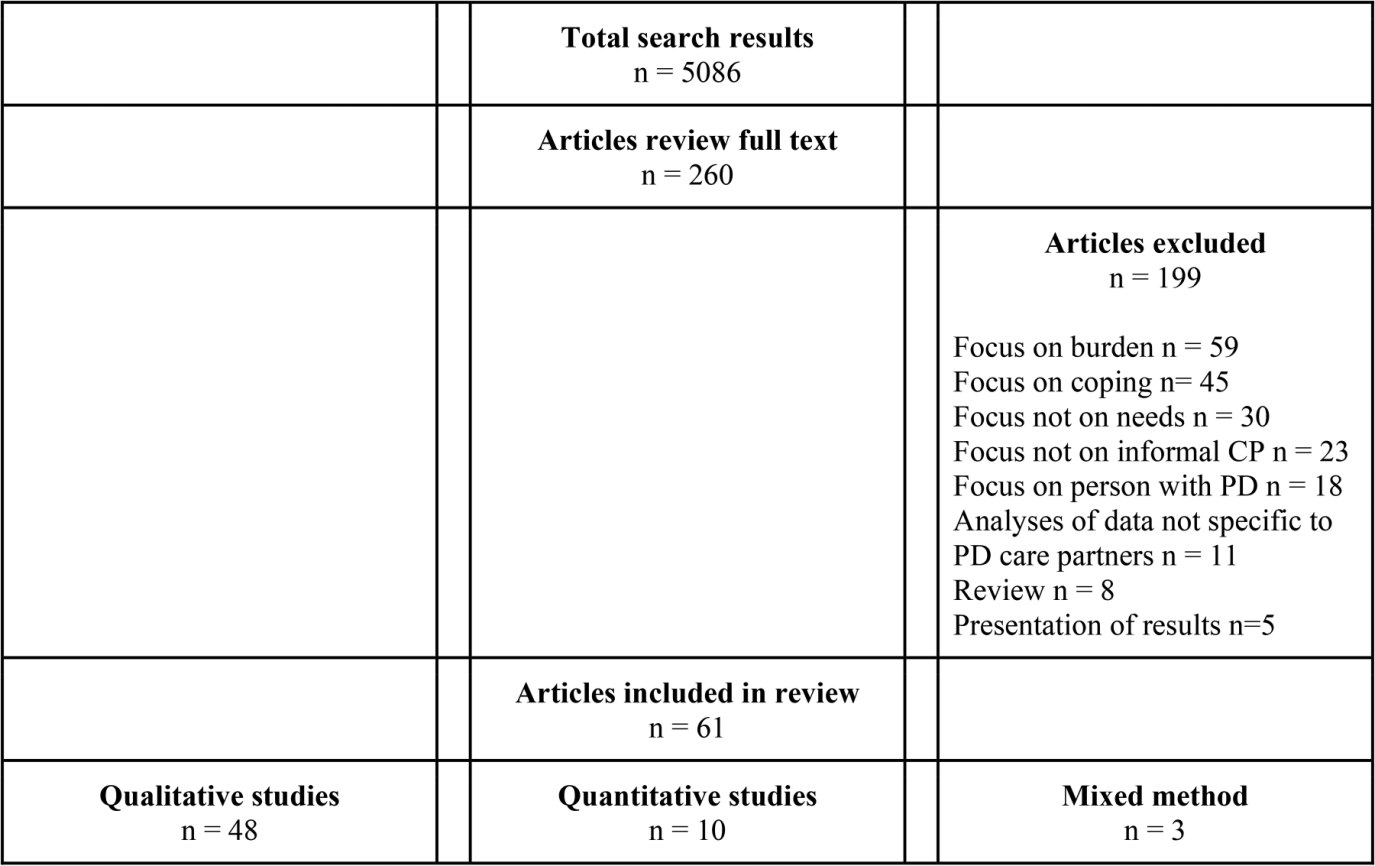
Search results on PubMed.

Sixty-one studies qualified for inclusion (see Figure 1); 48 qualitative studies, 10 quantitative studies and three mixed methods studies. The studies were conducted in the United States (16), the United Kingdom (11), Australia (6), Sweden (4), the Netherlands (5), Canada (4), Brazil (2), Germany (2), India (2), Norway (2), and one study each from China, Denmark, Italy, Korea, Malaysia, Singapore, and Slovenia. All studies were of observational, cross-sectional design.

The 51 studies with qualitative data were published between 2004 and 2024. The sample size of care partners in studies ranged from four to 36 for qualitative studies. Most qualitative studies collected data using individual interviews (n = 36), with some using focus group(s) (n = 4), survey (n = 2), dyadic interviews (n = 2), group interviews (n = 1) or a mix (n = 6). 27–100% of care partners identified as female. The mean age of the care partners ranged from 33–73 years. All studies scored eight or above out of 10 on the CASP. Qualitative study details are summarized in Table 1.

**Table 1.** Characteristics of qualitative studies and mixed method studies.

Study	Design	Care partners			Person with Parkinson's disease		CASP^15^
		Sample size	Female %	Age (Mean ± SD)	Eligibility criteria	Disease duration or time since diagnosis (Mean ± SD)	
Fekonja Z (2024) Slovenia^17^	Individual interviews	10	Not stated	Not stated	PD, not further specified	Not stated	9/10
Kapelle WK (2024) The Netherlands^18^	Individual interviews	16	56.3%	50 (8.0)	Young onset PD, diagnosed with idiopathic PD between 21–50 years	Not stated	10/10
Krieger T (2024) Germany^19^	Focus groups, paired interviews, individual interviews	29	75.9%	60.59 (9.64)	PD, not further specified	Not stated	9/10
Sharma P (2024) India^20^	Focus group	18	Not stated	Not stated	PD, according UK PD Society Brain Bank clinical diagnostic criteria	Not stated	9/10
White DR (2024) USA^21^	Individual interviews	13	100%	Not stated	PD, H&Y 2–4	Not stated	*8/10
Hjelle EG (2023) Norway^22^	Individual interviews	14	64%	73.07 (-)	PD, not further specified	8.57 (-)	10/10
Merritt RK (2023) UK^23^	Individual interviews	36	66.67%	Not stated	PD, not further specified	Not stated	9/10
Pigott JS (2023) UK^24^	Individual interviews	6	67%	60.2 (-)	PD diagnosis by specialist, cognitive impairment identified by clinician and symptoms recognized by participant	Not stated	9/10
Read J (2023) UK^25^	Individual interviews	11	91%	78 (-)	PD according UK PD Society Brain Bank clinical diagnostic criteria > 7 years, H&Y 4 or 5 during ‘ON’ state or disability indicated by score of 50% or below on S&E scale	17 (-)	9/10
Read J (2023) UK^26^	Individual interviews	17	59%	66 (-)	PD, not living in a care home, has capacity to provide informed consent to an interview, have life expectancy of more than six months, not atypical Parkinsonism	Not stated	9/10
Seshadri S (2023) USA^27^	Individual interviews	16	69%	68.3 (-)	PD, not further specified	7.5 (-)	9/10
Geerlings AD* (2022) The Netherlands^28^	Survey	141 (current care partners); 15 (former care partners)	68%; 80%	68.0 (7.8); 67.5 (10.7)	PD, not further specified	Not stated	8/10
Shahmoon S (2022) UK^29^	Individual interviews	16	50%	Not stated	PD, 1–5 years after DBS	Not stated	9/10
Bhasin SK (2021) India^30^	Individual interviews	10	100%	Not stated	PD, not further specified	Not stated	9/10
Jacob SA (2021) Malaysia^31^	Focus groups	4	Not stated	Not stated	PD, not further specified	6 (3.2)	9/10
Zhong X (2022) China^32^	Individual interviews	15	27%	49.1 (-)	PD, not further specified	10.8 (-)	10/10
Deutsch CJ (2021) Canada^33^	Individual interviews	12	67%	73 (-)	PD, active or present delusional thoughts	13.6 (-)	9/10
Rosqvist K (2021) Sweden^34^	Individual interviews	9	78%	Not stated	Idiopathic PD for >7 years, H&Y stages 4 or 5, have ON state and/or having a substantial need of help with ADL	Not stated	9/10
Cianfrocca C (2020) Italy^35^	Individual interviews	14	71%	Not stated	PD, MoCA >24, H&Y 2.5/3	Not stated	10/10
Duits A* (2020) The Netherlands^36^	Individual interviews	353	68%	68.7 (7.7)	PD, not further specified	Not stated	9/10
Jordan SR (2020) USA^37^	Survey, individual interviews	30	77%	68 (7)	PD, UK Brain Bank criteria for probable PD	9.17 (6.4)	9/10
McKeown E (2020) UK^38^	Survey, focus group	13	92%	Not stated	PD exhibiting ICBs related to dopamine therapy	Not stated	9/10
Armstrong MJ (2019) USA^39^	Individual interviews	20	55%	Not stated	PD with OFF periods	7.8 (4.7)	9/10
Lopes Nunes SF (2019) Brazil^40^	Individual interviews	20	80%	Not stated	PD, not further specified	Not stated	9/10
Padovani A (2018) Brazil^41^	Individual interviews	10	80%	Not stated	PD, not further specified	Not stated	10/10
Schwartz R (2018) USA^42^	Individual interviews	Not stated	Not stated	Not stated	PD, not further specified	Not stated	9/10
Boersma I (2017) USA^43^	Individual interviews	11	82%	65 (8.2)	PD, UK Brain Bank Criteria for probable PD and H&Y 2 or higher	Not stated	10/10
Habermann B (2017) USA^44^	Individual interviews	14	50%	72.13 (8.8)	PD, totally dependent on assistive devices for mobility, no dementia	12.18 (4.2)	9/10
Hurt CS (2017) UK^45^	Survey	18	67%	65.4 (5.1)	PD, not further specified	10.3 (6.0)	10/10
Bolland M (2015) Australia^46^	Individual interviews	15	73.33%	65.1 (13.3)	PD, not further specified	9.2 (6.5)	8/10
Carnett Martin S (2015) USA^47^	Individual interviews	23	65.22%	Not stated	PD, not further specified	Not stated	8/10
Hellqvist C (2015) Sweden^48^	Individual interviews, focus group	7	71%	Not stated	PD, idiopathic	Not stated	10/10
Thomas KE (2015) Australia^49^	Individual interviews	2	50%	Not stated	PD, not further specified	18 (11)	9/10
Barken R (2014) Canada^50^	Group interviews	Not stated	Not stated	Not stated	PD, not further specified	Not stated	8/10
Davis LL (2014) USA^51^	Individual and dyadic interviews	72	Not stated	Not stated	PD, not further specified	Not stated	10/10
Leiknes I (2014) (Norway)^52^	Individual interviews	9	66.67%	Not stated	PD, not further specified	Not stated	9/10
Van Rumund A (2014) The Netherlands^53^	Dyadic interviews	15	33%	Not mentioned	PD, MMSE < 18	11.4 (-)	10/10
Beaudet L (2013) Canada^54^	Individual interviews	10	60%	69.1 (5.99)	PD, moderate stage	11.6 (8.5)	8/10
Abendroth M (2012) USA^55^	Individual interviews, focus groups	20	70%	Not stated	PD, not further specified	Not stated	9/10
Tan SB (2012) Singapore^56^	Individual interviews	21	81%	Not stated	PD, idiopathic	Not stated	9/10
Duncan GF (2011) Australia^57^	Dyadic interviews	8	Not stated	Not stated	PD, not further specified	Not stated	9/10
Hounsgaard L (2011) Denmark^58^	Individual interviews	10	100%	65.8 (-)	PD, not further specified	3.7 (-)	9/10
McLaughlin D (2011) UK^59^	Individual interviews	26	65%	Not stated	PD, not further specified	Not stated	10/10
Van der Eijk M (2011) The Netherlands^60^	Individual interviews	20	75%	63.0 (1.8)	PD, not further specified	6 (5)	10/10
Hasson F (2010) UK^61^	Focus group	15	27%	Not stated	PD, deceased	Not stated	10/10
Giles S (2009) Canada^62^	Focus group	4 (-)	75%	54.25 (-)	PD, H&Y 2.5–5	Not stated	10/10
McCabe MP (2009) Australia^63^	Individual interviews	8	87.50%	67 (3.55)	PD, not further specified	9.31 (7.30)	8/10
Hudson PL (2006) Australia^64^	Individual interviews	21	71%	Not stated	PD, not further specified	Not stated	9/10
Habermann B* (2005) USA^65^	Individual interviews	20	Not stated	71.3 (-)	PD, not further specified	Not stated	9/10
Davey C (2004) UK^66^	Individual interviews	14	79%	69.9 (-)	PD, experienced repeated falls in the past 12 months	16.7 (-)	10/10
Birgersson AMB (2004) Sweden^67^	Individual interviews	6	33%	73 (-)	PD, not further specified	12.83 (-)	9/10

*: Mixed method study

(-): Not reported

CASP: Critical Appraisal Skills Programme^15^

ICB; Impulse control disorder

The 13 studies with quantitative data were published between 2003 and 2022. The sample size of care partners ranged from 12 to 324 for quantitative studies and from 20 up to 353 for mixed-method studies. One study made use of a validated questionnaire developed to measure care partner needs in patients with traumatic brain injury, the Family Needs Questionnaire (FNQ).^68,69^ The FNQ has 37 items on unmet needs scored as ‘yes’, ‘no’, or ‘partially’ within the following subscales: health information, emotional support, instrumental support, professional support, community support network, and involvement with care. No other studies made use of a validated or previously published questionnaire to measure care partner needs, although three studies devised a measurement instrument.^65,70,71^ The Caregiver Assistance Measure (CAM) rated 16 items on a Likert scale from 1 (assistance in this area is not important) to 3 (assistance in this area is very important) in three dimensions: the need for caregiving knowledge and skills, the need to find and use community resources, and the need to access personal assistance services.^65^ Another study asked participants to rate items on a Likert scale from 1 (no help is needed) to 4 (help needed all the time)^70^ and one study used preliminary, dichotomous data of the Parental Illness Impact Scale (PIIS).^71^

Overall, 55–85% of care partners participating in the quantitative studies identified as female. The mean time since diagnosis was mentioned in only three studies and ranged from nine to 12 years. The mean age of the care partners ranged from 64–71 years. All studies scored four out of 10 or below on the NOS. Details on the quantitative study characteristics are shown in Table 2.

**Table 2.** Characteristics of quantitative studies and mixed method studies.

Study	Measurement instrument	Care partners			Person with Parkinson's disease		NOS^16^
		Sample size	Female %	Age (Mean ± SD)	Eligibility criteria	Disease duration or time since diagnosis (Mean ± SD)	
Geerlings AD* (2022) The Netherlands^28^	Items on toppings for additional support	141	68%	68.0 (7.8)	PD, not further specified	Not stated	3/9
Zauszniewski JA (2022) USA^72^	Items on resourceful- ness scale	22	Not stated	Not stated	PD, not further specified	Not stated	2/9
Duits A* (2020) The Netherlands^36^	Items on need for support	353	68%	68.7 (7.7)	PD, not further specified	Not stated	2/9
Shin JY (2020) USA^73^	Items on information needs	54	59%	57.74 (16.9)	PD, not further specified	Not stated	2/9
Perrin PB (2019) USA^68^	The Family Needs Questionnaire	105 US care partners, 148 Mexican care partners	68.6%, 76.4%	68.73 (8.36), 53.66 (14.96)	PD, not further specified	Not stated	4/9
Sturm D (2019) Germany^74^	Items on topics of a psycho- educational group intervention	61	75%	70.54 (8.39)	PD, not further specified	12.05 (6.45)	4/9
Baik JS (2017) Korea^75^	Items on governmental policy on PD	151	55%	Not stated	PD, not further specified	Not stated	2/9
Olsson Y (2016) Sweden^76^	Items on access to healthcare services	66	70%	69.6 (8.2)	PD, not further specified	9.3 (6.0)	4/9
Lageman SK (2015) USA^77^	Items on caregiver- identified areas of need	66	80%	64.92 (10.91)	PD, not further specified	Not stated	1/9
Kristjanson LJ (2006) Australia^70^	Items on extent of importance of services	141	74%	66.2 (10.3)	PD, not further specified	9.4 (6.3)	3/9
Habermann B* (2005) USA^65^	Caregiver Assistance Measure (CAM)	20	85%	71.3 (-)	PD, not further specified	Not stated	4/9
Schrag A (2004) UK^71^	Parental Illness Impact Scale - PD	89	60%	Not stated	Young onset PD before the age of 40 years	Not stated	4/9
Parrish M (2003) USA^78^	Items on care partner needs as part of larger survey	324	84%	64 (-)	PD, not further specified	Not stated	1/9

*: Mixed method study

(-): Not reported

NOS: Newcastle-Ottawa Scale^16^

### Identified themes

Nine themes were identified from the analysis of the qualitative studies as shown in Table 3. Quantitative studies provided some supporting data for many but not all of the themes; no additional themes were identified from the quantitative studies.

**Table 3.** Care partner needs: themes and subthemes identified from the literature.

Theme	Example excerpts and quotes	Qualitative studies (n)	Quantitative studies (n)
**The need for information**		**30** ^18,19,22–26,28,31,32,34,37,39,40,43–45,48,49,54,56–62,66,67,79^	**8** ^28,53,65,68,71,73,74,77^
PD prognostic information for future planning	*“When considering the future, patients and care partners had several questions related to how their PD would change over time and how quickly it would change (i.e., speed of PD illness trajectory). These questions reflected a desire for information that would address their personal experiences compared to an expected PD trajectory and/or the experiences of others.”* ^37^		
PD treatment	*“Many patients and informal caregivers expressed a desire to be actively involved, and to be able to participate in shared decision-making with their professional caregivers. However, they identified a current lack of information.”* ^60^		
Care partner education resources	*“Information from health and social care professionals was felt to be patchy and not forthcoming, which led carers to seek information on the internet, from other people with the condition or elsewhere –– Yes, we would use the internet a good bit and also there are a lot of leaflets that they [PDUK] publish. Go round finding out of the people (PDS group) what drug they are on and if I see any improvement. But then I am able to talk to them.”* ^59^		
Financial	“*And I am really concerned. Will we outlive our money? I think financial education is about how you go about knowing. You know, nobody can predict, per se, but a year ago the doctor told us - one of the doctors - said my husband would be in need of nursing care.”* ^43^		
Home adaptation	*“There were difficulties accessing information about entitlements, benefits and equipment. Most were not aware what help was available, to what they were entitled or whom to contact and were unsure how to complete the necessary forms, viewing the process as very time-consuming. This resulted in some not even claiming for support with others only finding out by chance what was available.”* ^59^		
**The need to be heard**	*“Over the period of time it had made me feel that I am just a caregiver, I need to be heard!”* ^30^	**12** ^18,19,21,22,30,35,36,43,46,56,60,79^	**1** ^28^
	“*It's always him (neurologist) and Jack, and he doesn't ask me how I feel…* *it didn't occur to me that he should ask me, but I guess the primary care as far as he's concerned is Jack.”* ^56^		
**PD healthcare**	*“Some caregivers suggested that the healthcare system could be improved if a more integrated approach to providing PD services were adopted.”* ^56^	**16** ^19,20,24,26,34,45,46,50,52,53,56,57,59,62^	**7** ^36,70,71,74–77^
	“*Former carers agreed that they should have been provided with a more integrated care package.”* ^61^		
**Emotional support**	*“I couldn't say I have received it (support) in any way…* *you kind of…* *you just get on with it…* *nobody really knows, actually knows how bad things are…* *you do just feel isolated and you have no-one to turn to.”* ^46^	**34** ^17–19,22,24,26,28,29,31–33,37,38,40–48,50,51,54–56,58,60–62,64,67,79^	**8** ^28,36,65,71,72,77,78^
	“*In addition, many carers experienced a sudden loss of contact from health and social care professionals and social support agencies, resulting in loneliness.”* ^61^		
**Daily living (assisted living and home adaptations)**	*“Another caregiver frequently mentioned a need for assistance with activities of daily living, usually in the morning.”* ^64^	**12** ^17,25,34,35,37,43,50,54,57,62,64,66^	**3** ^65,70,71^
	“*For the informal caregivers who did not live together with the patient, the starting point to discuss a move to a residential care facility was clearer, when it became obvious that the support provided by the home health care and social services was not sufficient.”* ^34^		
**Financial support**	*“So I need to make sure that I keep working to help pay for all the bills and cause the insurance is through my work.”* ^43^	**12** ^18,19,32,37,38,43–45,56,59,62,64^	**3** ^28,70,74^
	“*When will I stop working? When will my partner stop working? (…) We need to have money for a care home for home adjustments. Those are pretty hard time.”* ^18^		
**Skills for providing care**	*“I have no experience of nursing, and to help someone to dress, you maybe do it backwards.”* ^67^	**6** ^27,35,43,61,66,67^	**7** ^28,36,65,70,71,77,78^
	“*Yeah, the ultimate is me not being able to be trained enough because I am not medical you know? I don’t have any medical training to care for him.”* ^43^		
**Care partner physical well-being**	*“Both carers identified a need for physical strengthening exercises to increase their ability to complete manual handling tasks.”* ^49^	**8** ^18,30,43,49,56,58,59,66^	**(-)**
	“*I worry that if I will fall sick. I cannot get sick because I’m a caregiver….the main thing I’m really worried about is my health. I have to keep fit and continue to work.”* ^56^		
**Respite care**	*“As you can see, I don’t want to go on holiday because if I go on holiday then I will feel guilty because I will leave my mum and maid behind. I don’t want to get somewhere and then feel bad.”* ^56^	**26** ^18,24,28,34,43,44,56,57,59,61,66,67,79^^−,^^20,23,29,30,32,33,40,42,46,51,52,63,64^	**6** ^28,36,65,70,77,78^
	“*I would like to have some time to the grocery store or go to the library things like that. Because she can’t transfer, I can’t leave her.”* ^44^		

### The need for information

*Qualitative results.* The need for information was identified in 30 qualitative studies, and was primarily attributed to suboptimal communication from health care providers, including neurologists, general practitioners and PD specialist nurses.^18,19,22–26,28,31,32,34,37,39,40,43–45,48,49,54,56–62,66,67,79^ The information needs can be subdivided into five subthemes: prognostic information for future planning, PD treatment, care partner education resources, finance, and home-adaptation. Exemplary quotes illustrating each subtheme are shown in Table 3. Notably, several qualitative studies alluded to the imbalance in power dynamics between care partners and some healthcare providers, resulting in a lack of open communication. Some of these care partners who felt they were ill-informed resorted to unreliable internet sources which elicited fear in care partners provoked by inaccurate prognostic information.^62^ Care partners reported insufficient information on available healthcare resources.^45,54,61^ Those who were more fully aware of the currently offered services reported having to actively seek out and inquire instead of being informed.^61^

*Quantitative results.* The need for information was addressed in eight quantitative studies.^28,53,65,68,71,73,74,77^ Twenty US care partners filled out the CAM questionnaire and rated ‘information about the illness’ and ‘medical treatment advice’ relatively high with scores of 2.66 and 2.20 on a Likert scale from 1 to 3.^65^ In contrast, only 20% of a Dutch cohort comprising 141 care partners indicated ‘information about PD and medication’ as a need.^53^ In descending order, ‘information on finding and applying for home care services’ was rated 1.89/3, ‘information on community resources’ was rated 1.75/3, ‘help getting medical equipment and supplies 1.70/3’, ‘information on home modification 1.70/3’, ‘help getting affordable home modifications 1.63/3’, ‘information on applying for financial assistance 1.45/3’, and ‘information on finding transportation 1.25/3’.^65^ Information about how to receive social and financial support’ was addressed as a need by 23% of care partners and 18% addressed ‘information about possible strategies for dealing with stress’ as a need.^53^

Twenty of 89 US care partners who were children of someone with young onset PD answered ‘yes’ when asked whether ‘having more information would lead to less feelings of uncertainty and insecurity’.^28^ 13.6% of 66 US care partners reported ‘patient education about diagnosis’ as an area of need and 60.6% identified ‘planning for the future’ as an area of need in another study.^71^ Sixty-one German care partners for persons with PD with a mean disease duration of 12 years were asked about relevant topics for a psychoeducational group intervention and 41% deemed ‘information on PD and treatment’ a relevant topic,^77^ suggesting that needs in this domain vary across regions or groups.

Out of 353 Dutch spouses of persons with PD, 49.7% reported needing support for finding their way in health care.^74^ In another study 57.4% of 61 German care partners deemed information on services for the patient a relevant topic and 50.8% deemed information on services for relatives a relevant topic for a psychoeducational intervention.^77^ Fifty-four US care partners indicated their need for information on several topics and rated ‘managing their emotional and physical stress’ with 46.3% as the highest identified informational need.^73^

### The need to be heard

*Qualitative results.* Spousal care partners expressed the need for their feelings or thoughts to be heard by others in 12 qualitative studies.^18,19,21,22,30,35,36,43,46,56,60,79^ An Italian care partner expressed a yearning to share her sentiments: “I think that sharing my fears and my thoughts could help me in understanding myself better”.^36^ Similarly a Chinese care partner who indicated a need for a communication platform for care partners to get together: “*I even thought of doing it myself, setting up a QQ chat room (online platform) for patients with PD and their families*”.^35^ Some US female care partners addressed a paucity of regard for their wellness and attributed it to their gender role: “*Only two people have asked how are you? To me. How am I? It just doesn’t happen. How is Steve? We, just as women, are taught anyway not to talk about ourselves and to be the focus of attention. And it's like if somebody asks me: how are you? They just work their way into my heart, that's what I need*'’.^43^

*Quantitative results.* In a Dutch study with 141 care partners, 38% reported ‘learning to communicate based on your own needs and wishes as a need.^28^

### Parkinson's disease healthcare

*Qualitative results.* Care partners expressed the need for better organization of multidisciplinary care services for PD.^19,20,24,26,31,34,45,46,50,52,53,56,57,59,61,62^ “*I keep saying we need better services and solutions. What we have now is not enough and it's all over the place. Hospitals could package services into one center with one slot of time where you can do speech (speech therapy), physio (physiotherapy) and all that”*.^56^ Care partners from a UK study uttered their frustrations about the inadequate accessibility to services: “*Many caregivers described a consuming search, going round in circles to access services or getting caught up in a lot of bureaucracy (Caregiver 14)*’.^24^ In areas that do offer complementary care services, the lack of coordination and affordability add another layer of barrier. A Canadian care partner spoke of their need for a solution: “*You know for people who can’t afford it and just yeah, if it was all in one building that would be amazing and if we didn’t have 50 million different places and like try to figure out if they’re able to do it and care for the people”*.^62^

*Quantitative results.* Seven out of 13 studies reported on PD healthcare needs.^36,70,71,74–77^ An early onset diagnosis was reported by 15.2% of 66 US care partners as an area of need.^77^ Three studies reported on access to health care: 41% of 58 Swedish care partners reported insufficient access to health/social services during the past six months versus 9% of an age-matched control group,^76^ out of 353 Dutch spouses of persons with PD 31.9% needed support for dealing with health care contacts,^36^ and 48.3% of 89 children of young onset PD answered ‘yes’ when asked ‘would help to be able to talk to local services about help provided to parent’.^71^ Care partners also reported a need for access to palliative care in a study of 141 Australian care partners with a disease duration of almost 10 years rated information access to palliative care was rated 3.3 on a Likert scale from 1 to 4.^70^ A Korean study with 151 care partners reported that 46% thought that the government should politically take into consideration a family support system, 25% thought that the government should take into consideration reinforcement of education for PD and PD treatments, and 22% that the government should take into consideration support training of specialized institutions and specialists.^75^ Sixty-one German care partners were asked about relevant topics for a psychoeducational group intervention: 59% identified a need for information on care as a relevant topic, 57.4% information on services for the patient, and 50.8% on services for relatives.^74^

### Emotional support

*Qualitative results.* Emotional support can be divided into emotional support from social networks and emotional support from the healthcare practitioner(s). Care partners’ need for emotional support from social networks may not be sufficiently met, as reported by 21 of the qualitative studies.^17,22,24,26,28,29,31–33,38,40–42,45,50,51,55,56,61,64,79^ For example spousal care partners included in an Australian study reflected on the lack of emotional support from their children and extended social network: “*I feel angry and bitter and frustrated because even just a phone call to say how you are doing mum, or would you like us to take dad out for the afternoon and you have a break, you know. Something, even if it was just a conversation (No.177)”*.^64^ A care partner in a US study also spoke about the lack of availability of support systems having a negative impact on their wellbeing. They explicitly stated a need for empathy and emotional support.^55^

Care partners’ need for emotional support from their healthcare providers was identified in 23 studies included in this review.^18,19,22,24,26,31–33,37,40,43–48,54,58,60–62,67,79^ For example, Swedish care partners emphasized the importance of receiving emotional support and validation from PD specialist nurses, such as being recognized for their relentless efforts of caring for the person with PD or offering companionship to care partners when they are feeling vulnerable.^54^ Care partners’ need for emotional support may remain even after the passing of the person with PD; care partners in a UK study reported experiencing a loss of contact from all of their healthcare providers after the passing of their spouses which worsened their feelings of loneliness.^61^

*Quantitative results.* In a US study with 324 care partners, the second most common need identified was emotional support (by 83%). 38% reported the informal help and social support they were receiving was far less than they needed and 12% responded ‘not at all’ when asked if they knew where and how to request help from others.^78^ 22% of 353 Dutch spouses needed support for maintaining social contacts. They also needed support for dealing with losses (42.2%), dealing with uncertainty and feelings of shame and guilt (23.6%), and for intimate relations and sexuality (16.5%).^36^ In a Dutch study with 141 care partners, the most frequently reported emotional support need was ‘dealing with a changing relationship with the person with PD (26%)’, followed by ‘taking care of myself as a caregiver (25%)’, ‘dealing with changing relations and reactions from environment (19%)’, and ‘accepting my own/new role as an informal caregiver (15%)’.^28^ ‘Adjustment to the diagnosis’ was reported as an area of need by 19.7% of 66 US care partners. Other areas of need related to emotional support identified were ‘stress management’ 37.9%, ‘caregiver stress’ 45.5%, and ‘relationship changes’ 59.1%.^77^ 42.7% of 89 US children of young onset PD answered ‘yes’ when asked ‘helps to have contact with people in similar circumstances e.g., support groups’.^71^ Similarly ‘a chance to talk with other caregivers’ received an average rating of 2.35/3 by 20 US care partners that filled out the CAM questionnaire.^65^

Regarding relevant topics for a psychoeducational group intervention, 61 German care partners 59% identified ‘stress management’ a relevant topic, 50.8% ‘receiving social support’, 50.8% ‘coping with anxiety and depression’, 42.6% ‘promoting well-being’, 39.9% ‘sharing experiences with other relatives’, and 34.4% ‘expressing needs and desires’.^74^

Two studies reported on emotional support from the healthcare practitioner: 23.6% of 89 children of young onset PD answered ‘yes’ when asked ‘would like the opportunity to have counselling’^71^ and of 20 US care partners that filled out the CAM questionnaire, they rated ‘individual counselling to help me cope’ 1.75/3.^65^ One online survey study with 22 care partners reported a ‘severe to very severe’ need for resourcefulness training compromising three social (help-seeking) and five personal (self-help) resourcefulness skills intended to support emotional health among other aspects of well-being.^72^

### Daily living (assisted living and home adaptation)

*Qualitative results.* As the person with PD's health deteriorates, care partners may find themselves struggling with the increasing responsibilities and eventually the need to admit them into an assisted living facility: “*And I have spoken to my dad about it and I have said to him – I have got to put my kids first, same as you would have put me first, you have to go (1071)”.*^25^ The need for guidance related to daily living adjustments was identified in 12 studies.^17,25,34,35,37,43,50,54,57,62,64,66^ For example, a care partner explained: “*It would be really nice to have a person who would come in but with a complete understanding of all his needs. The caregiver who comes in now, she doesn’t know enough about him to do that”.*^35^ Information on how to apply for housing adaptations and how to get occupational therapists involved in the process were also identified as needs.^34^ A spousal care partner participant in another US study reported being unsure when they should start considering assisted living: *“What does everybody else do? How do you know when it's time to say you need somebody full-time in the evening and daytime, when do we need to consider assisted living or nursing homes? I don’t know if there are any trigger points for that because he's not there yet, I don’t think”*.^37^

*Quantitative results.* There are three studies that addressed this theme, but in contrast to the qualitative studies they focused on the services and care needed rather than guidance on choosing or accessing these. On the CAM questionnaire, ‘assistance with the household chores’ was rated 1.90/3.^65^ 69.7% of 89 children of young onset person with PD answered ‘yes’ when asked ‘think more help (e.g., meals on wheels, physiotherapy) should be provided’ and 55.1% answered ‘yes’ when asked ‘outside help available to help care for parent with PD’.^71^ 141 care partners from Australia for persons with PD with a mean disease duration of almost 10 years rated the following needs: ‘equipment for daily living’ (3.00/4), ‘home support services day’ (2.37/4) and ‘home support services night’ (1.93/4), and’ reliable ongoing dependable support workers (2.84/4).^70^

### Financial support

*Qualitative results.* Twelve studies included in the review identified the financial issues faced by persons with PD and their informal care partners.^18,19,32,37,38,43–45,56,59,62,64^ For some families, their savings were threatened due to the cost of medications and other treatments. For example, a care partner from a US study identified their need for a financial advisor: “*I could use someone to help us look at - Ok, here is how much money you have (…)- you can afford these, and you probably can’t afford these - That kind of thing will be helpful. - female, 63”*.^43^ Another US care partner described feeling concerned about whether or not their retirement savings will suffice and a similar need for financial education pertaining to PD: “*I was talking to a social worker who said there are real problems down the road financially if you have assisted living and I need to know more about that to prepare financially”*.^37^

*Quantitative results.* Australian care partners rated financial assistance for care 2.72/4.^70^ Almost half (45.9%) of 61 German care partners deemed receiving financial support a relevant topic for a group intervention.^74^ Out of 141 Dutch care partners, 23% identified ‘information about how to receive social and financial support’ as a need.^28^

### Skills for providing care

*Qualitative results.* Informal care partners in six qualitative studies reported wanting to learn how to use PD-specific equipment properly in order to physically assist their care recipients in daily activities and how to strategically manage falls.^27,35,43,61,66,67^ For example, a US care partner stated: “*Maybe something on teaching caregivers how to deal with that. How to make them fall. I mean you can’t catch them, cause you’ll hurt yourself. But helping them fall so they don’t hurt themselves or you. Or anything else, right? Or any of the first aid. I mean I have to be first aid, CPR trained. But they don’t teach you some of that stuff for specific issues”*.^43^ An Italian care partner indicated wanting to receive first aid training: “*My father is old, and I have the fear of what to do if something happens at home, for example, a heart attack or a bad fall or if he loses consciousness”*.^35^

*Quantitative results.* Care partners listed skills needs for providing care in a total of seven quantitative studies.^28,36,65,70,71,77,78^ Of 353 Dutch spouses of persons with PD, 34.8% needed support for recognizing body signals and taking these into account.^36^ A US study reported that of 324 PD care partners, the third most common problem (32%) was the need for behavioral management.^78^ 31.8% of Dutch care partners reported emotional changes as an area of need and 27.3% reported personality and behavior issues that they felt unequipped with the skills to address.^36^ 42% out of 141 care partners identified recognizing signals in the person with PD (e.g., depression, dementia, hallucinations) as a need, 26% dealing with psychosocial consequences in PD (e.g., anxiety, depression), and 20% developing skills for dealing with stressful situations.^28^

39.3% of 89 children of persons with young onset PD answered ‘yes’ when asked ‘would help to have training in giving practical care’,^71^ ‘training to improve my caregiving skills’ was rated 2.25/3 on the CAM questionnaire by 20 US care partners,^65^ information about how to provide care was rated 3.31/4 by 141 Australian care partners.^70^ Communication skills were addressed in several quantitative studies: family communication earned an average rating of 2.5/4 by 141 Australian care partners, 41% of 61 German care partners deemed improving communication with the patient a relevant topic for a group intervention,^77^ and ‘learning how to talk with health professionals more effectively’ was rated 2.1/3 on the CAM questionnaire by US participants.^65^

Other areas of skills needs endorsed by 66 US care partners were lifestyle changes (e.g., safety, travel, driving, 71.2%), wellness strategies (e.g., exercise, nutrition, spirituality, 57.6%) and thinking strategies (e.g., attention, memory, problem solving, 57.6%).^77^

### Care partner physical well-being

*Qualitative results.* Eight studies included care partners who felt the need to take a step back and take care of their physical health.^18,30,43,49,56,58,59,66^ For example, an Irish care partner explained: “I’ve *found that catching him strains my arms and shoulders. My right hip is a bit arthritic and that went completely about six years ago. Then I had a slipped disc about three years ago and now I have this flipping shoulder. I put it down to catching him, I’m not going to get him up, I’m going to have to get help”*.^66^ Many care partners in the same study reported being troubled with pain and strain from physically supporting the person with PD on a daily basis.^66^

*Quantitative results.* There were no quantitative results related to care partner physical well-being.

### Respite care

*Qualitative results.* Care partners in a total of 26 studies included in this review identified their need for respite care.^18,20,23,24,28–30,32–34,40,42–44,46,51,52,56,57,59,61,63,64,66,67,79^ For example, a care partner in a Swedish study illustrated her need for some undisturbed sleep: “Last *night I did not sleep more than two hours at the time, maybe just one. You have to make sure you get to sleep for another little while, so it will get better again”*.^24^ Although many care partners recognize their need for respite care and may have access to this service, care partners expressed difficulty leaving their care responsibilities because of the guilt they experience leaving the person with PD. A care partner in the same Singaporean study described, “*As you can see, I don’t want to go on holiday because if I go on holiday then I will feel guilty because I will leave my mum and maid behind. I don’t want to get somewhere and then feel bad”*.^56^ Although they are aware of the importance of having time away, many care partners have reported not being able to do so because of the person with PD's dependency on them.

Many informal care partners juggle multiple roles and are overwhelmed with competing responsibilities.^34,43,56^ A Singaporean care partner shared their need to attend to the caregiving of their elderly mother while taking care of their husband who has PD: “*I also have to take care of my aged mother, take care of my mum and take care of my husband. My mum also sometimes gives me problems… she is also sick”*.^56^

*Quantitative results.* Respite care was featured in six quantitative studies.^28,36,65,70,77,78^ It was the most common need reported in a study of 324 US care partners (by 85%)^78^ and the most common need reported in a study of 141 Dutch care partners (by 44%).^28^ Out of 353 Dutch spouses, 34% needed support for balancing rest and activity and 18% needed support for adjusting personal interests and ambitions.^36^ On the CAM questionnaire, ‘someone to come and stay in the home so I can get some time for myself’ was rated 1.56/3 by 20 US care partners.^65^ Care partners rated respite during the day as 2.33/4 and during the night as 1.8/4.^70^ In just one study from the US, 22.7% of 66 care partners said that work related issues were an area of need.^77^

## Discussion

We identified nine themes of care partner needs in our review, all from the qualitative literature. The quantitative literature was sparse, and had significant methodological shortcomings. We will discuss these findings and implications for future research.

A major theme emerging from our review concerned ‘information’, which spanned various topics (see Table 3). Information on each topic, provided by healthcare practitioners, is needed to inform care partners and this need scored highest on the CAM questionnaire.^65^ We also identified ‘skills’ related to caregiving including skills for dealing with non-motor symptoms, general caregiving skills, communication skills with either the persons with PD or healthcare practitioners and how to deal with logistical problems as another important category of information needs. ‘'Skills” as an important need was supported by the quantitative literature; also being highly rated on the CAM questionnaire^65^ and being the third highest ranked need in another study.^78^ Both information and skills needs could potentially be met with relatively low cost interventions and could be cost-effective, enabling care partners to identify and address issues earlier before they lead to significant morbidity for the care recipient, and reducing care partner burden. Although major categories within these needs are identified, the specific disease-related information and skills needed are not described extensively. Further, information on when these needs are most commonly encountered in the PD disease process is lacking.

Across all needs, the need for ‘respite care’ was ranked third according to the number of qualitative studies identifying it as a need and it was the most reported need in two quantitative studies,^28,78^ though it scored lower in others.^36,65,70^ Respite was described as providing both personal time off for the care partners and time to take care of other responsibilities. Potential contributors to the need for respite, for example other responsibilities, being the only care partner, and work, should be studied more in depth. Care partner respite in PD is positively associated with quality of life, and given the relationship between care partner well-being and respite, these findings suggest that provision of respite services should be a priority for health care systems and agencies.^80,81^

Family and friends can also be a source of respite for care partners. However, a lack of appreciation for the burdens borne by care partners was a commonly cited perception, and may contribute to a lack of offers of help from these informal sources. Supporting care partners in reaching out for such support where available could be an effective intervention by health care teams, as could advocacy efforts in the PD community highlighting the burden on care partners and the need for respite. Such interventions can also contribute to emotional support, which was identified as the second highest need in one study.^78^

Related to care partner support, ‘the need to be heard’ was mentioned in only one quantitative study,^28^ and ‘care partner physical well-being’ was not addressed at all in quantitative studies, yet they were both mentioned as major concerns in qualitative studies.

The theme ‘daily living’ was identified in 12 qualitative studies and was addressed in three quantitative studies. Though it did not score high on the CAM questionnaire,^65^ other studies outlined higher needs in this theme, yet again more specific needs (household chores, support workers, home support, etc.) and associations to this theme were not studied.^70,71^ It is clear that care partners need more assistance with how to anticipate the need for and obtain appropriate additional support for the care of the person with PD.

‘PD healthcare’ was deemed important in qualitative data, but quantitative data available on this theme seemed to portray moderate need on this subject, with half of the care partners in need of help in finding their way in PD healthcare^36^ and relatively low scores on the CAM questionnaire regarding this theme.^65^

The least frequently mentioned themes in qualitative research were ‘skills for providing care’, mentioned in six studies, ‘care partner physical well-being’ mentioned in eight studies, and ‘financial support’ and ‘the need to be heard’ both mentioned in 12 studies each.

The majority of data found were from qualitative studies. Data from qualitative studies lend themselves well to describe the range of experiences of participants and their attendant needs. We used these descriptive data to create a logical narrative to frame the care partner needs in PD. Quantitative data can inform us regarding the prevalence and relative importance of specific needs and their associations. All quantitative data found could be placed in the framework of needs created from the qualitative data. Because quantitative data were scarce and because individual quantitative studies did not cover the range of care partner needs demonstrated in the qualitative literature, we were unable to clearly identify the most prevalent care partner needs. Similarly, the quantitative data available do not allow us to identify a timeline of needs in the course of PD. Quantitative studies identified were often not primarily focused on care partner outcomes and were of poor methodologic quality. The low scores on the NOS^16^ were largely due to the failure of representativeness and selection of the cohort and because a validated measurement instrument was not used.

Only one study^68^ used an established tool for measuring care partner needs, although the FNQ was originally developed for addressing care partner needs in traumatic brain injury.^69^ One study made use of a questionnaire specifically designed to measure care partner needs, the CAM, although this scale was developed for use in the same study and has not been validated, nor been used in other studies to our knowledge. Despite these limitations, the CAM was the broadest instrument used in the studies included in this review.^65^ It highlights most of the nine themes found in this review with the exception of ‘care partner physical well-being’ and ‘skills.’ There are several studies that use questionnaires that include items on care partner needs but also address other topics.^28,36,77^ Eleven items on the prevalence of caregiver needs were part of a larger survey in another study, addressing ‘information’, ‘PD healthcare’, ‘emotional support’, ‘skills’, and ‘respite care’.^77^ The need for support and received support for several themes were covered in two online surveys.^28,36^ These studies leave a number of the themes we identified unaddressed; all remaining quantitative studies made use of custom surveys with few items addressing care partner needs.

Potentially useful measurement instruments for future quantitative studies in this field can be identified from studies not included in our review. For the framework for our review, we used the SPUN-SF, a validated questionnaire on care partner needs for persons with cancer, due to its extensive coverage of themes. The SPUN-SF contains 26 items on information (6 items), the future (3 items), work and finance (5 items), healthcare access and continuity (5 items), personal needs (4 items).^13^ We identified two themes that were not represented in the SPUN-SF: ‘care partner physical well-being’ and ‘respite care.’ Therefore, modifications would be called for before adaptation in PD care partner needs research. At least two studies^82,83^ not covered in our review (due to not focusing on needs) have used the Belastungsfragebogen Parkinson angehörigen–kurzversion (BELA-A-k), that addresses the needs of care partners of people living with PD specifically. BELA-A-k is a 15-item questionnaire published in German, Dutch, and French which asks care partners to score the themes ‘achievement capability/physical symptoms,’ ‘fear/emotional symptoms,’ ‘social functioning,’ and ‘partner-bonding/family’ on a 5-point Likert scale for both bothersomeness and need for help.^84^

Other questionnaires designed to address care partner needs do exist,^85^ but we are unaware of other needs questionnaires specific to PD care partners. Quantitative research in care partner needs in PD could benefit from a more comprehensive yet dedicated instrument that is informed by the breadth of qualitative research in this field.

Despite the large number of qualitative studies regarding care partner needs in PD, many questions remain relatively unexplored. The majority of care partners of studies in this review were of female gender. Gender differences in care partner needs were rarely investigated in quantitative studies, but other research suggests the potential for importance differences; informal caregiving resources have been found to be less available for women than men living with PD.^86^ Gender disparities in care partner needs should therefore be investigated in future research.

Most studies were carried out in Western countries and conclusions of the review may not be generalizable to other cultures or to countries with non-similar healthcare systems. One quantitative study did investigate differences in care partner unmet needs in PD in Mexico and the US and concluded that care partners in the US were at increased risk for poor emotional and community support compared to care partners in Mexico.^68^ More emphasis on cross-cultural differences and the effect of healthcare system differences in care partner needs should be considered for investigation in future research.

Care partners in quantitative studies were either spouse, family members, children or this was not clearly stated. Care partner needs and the association with their relation to the person with PD were not investigated, although known relationships between care partner / recipient relationships, burden, and quality of life suggest that needs may indeed differ.^87–89^

The fulfilment of the care partner role was described in many studies; whether they had other care partners helping out, home assistance or were still working besides their caregiving role. Associations with the scope of the caregiving role were not tested in quantitative studies, and it's diversity raises questions how care partner needs could be affected as for instance job and family demands are associated with care partner strain.^90^

Advanced therapies for PD, as deep brain stimulation (DBS), and care partner needs were the focus of only one qualitative paper.^29^ The effect of DBS on care partner burden has been studied,^91,92^ but the effect of DBS or other advanced therapies on care partner needs still remains an area of interest.

To our knowledge, this is the only systematic review that has explored care partner needs in PD to date. Previous reviews rather focused on care partner experiences, partially covering care partner needs, and included qualitative literature only.^93,94^ Care partner burden has been studied to a greater extent in both cross-sectional and longitudinal studies, but there is a conceptual difference between needs and burden, even though they are two intricately interwoven topics. Burden can arise from unmet needs but could also arise unrelated to any need, as being a care partner is a demanding task itself. Conversely, needs arise from burden whether already experienced or foreseen, and addressing them could therefore alleviate burden when addressed. Thus understanding the needs of care partners and how they evolve across the journey of PD is critically important.^5^

An important limitation of this review is that it includes English literature only. We have seen variation in needs across studies reflecting differences between populations, which may be even greater if non-English languages were included. Another limitation was that the prevalence of the needs identified cannot be fully elucidated given the limited number and low quality of the quantitative studies found.

This review provides an overview of what is currently known about care partner needs in Parkinson's disease and highlights important gaps in quantitative literature. More studies of mostly quantitative design are needed to identify and understand the relative frequency of care partner needs and when to expect these needs in the normal progression of PD. Validated measurement tools for care partner needs in PD are needed to facilitate this work. Knowledge emerging from such studies will be critical to appropriately target resource development and service delivery to help care partners in their important and indispensable role in PD.

## Supplemental Material

sj-docx-1-pkn-10.1177_1877718X251344066 - Supplemental material for Care partner needs in Parkinson's disease: A systematic review of qualitative and quantitative data Supplemental material, sj-docx-1-pkn-10.1177_1877718X251344066 for Care partner needs in Parkinson's disease: A systematic review of qualitative and quantitative data by Max Hulshoff, Christine Sun, Elaine Book, Caroline Tanner, Nabila Dahodwala, Brenda Reynolds, Heather Boon and Connie Marras in Journal of Parkinson's Disease

## References

[1] Lum HD Jordan SR Brungardt A , et al. Framing advance care planning in Parkinson disease: patient and care partner perspectives. Neurology 2019; 92: e2571–e2579. 10.1212/WNL.0000000000007552 PMC6556088 31028124

[2] Martínez-Martín P Forjaz MJ Frades-Payo B , et al. Caregiver burden in Parkinson's disease. Mov Disord 2007; 22: 924–931; quiz 1060. 17238193 10.1002/mds.21355

[3] Parashos SA Maraganore DM O'Brien PC , et al. Medical services utilization and prognosis in Parkinson disease: a population-based study. Mayo Clin Proc 2002; 77: 918–925. 12233925 10.4065/77.9.918

[4] Kalia LV Lang AE . Parkinson's disease. Lancet 2015; 386: 896–912. 25904081 10.1016/S0140-6736(14)61393-3

[5] Hulshoff MJ Book E Dahodwala N , et al. Current knowledge on the evolution of care partner burden, needs, and coping in Parkinson's disease. Mov Disord Clin Pract 2021; 8: 510–520. 33981783 10.1002/mdc3.13201 PMC8088088

[6] Mosley PE Moodie R Dissanayaka N . Caregiver burden in Parkinson disease: a critical review of recent literature. J Geriatr Psychiatry Neurol 2017; 30: 235–252. 28743212 10.1177/0891988717720302

[7] Aamodt WW Kluger BM Mirham M , et al. Caregiver burden in Parkinson disease: a scoping review of the literature from 2017–2022. J Geriatr Psychiatry Neurol 2024; 37: 96–113. 37551798 10.1177/08919887231195219 PMC10802092

[8] Prizer LP Kluger BM Sillau S , et al. The presence of a caregiver is associated with patient outcomes in patients with Parkinson's disease and atypical parkinsonisms. Parkinsonism Relat Disord 2020; 78: 61–65. 32736164 10.1016/j.parkreldis.2020.07.003

[9] Schara H Johnson T Brungardt A , et al. Living with dementia: care partner needs and outcomes of a dementia support program in primary care. Gerontol Geriatr Med 2022; 8: 23337214221129466. 36275412 10.1177/23337214221129466 PMC9583199

[10] Hoseinpour F Ghahari S Motaharinezhad F , et al. Supportive interventions for caregivers of individuals with multiple sclerosis: a systematic review. Int J MS Care 2023; 25: 266–272. 37969907 10.7224/1537-2073.2022-083 PMC10634599

[11] Moher D Shamseer L Clarke M , et al. Preferred reporting items for systematic review and meta-analysis protocols (PRISMA-P) 2015 statement. Syst Rev 2015; 4: 1. 25554246 10.1186/2046-4053-4-1 PMC4320440

[12] Carroll C Booth A Cooper K . A worked example of “best fit” framework synthesis: a systematic review of views concerning the taking of some potential chemopreventive agents. BMC Med Res Methodol 2011; 11: 29. 21410933 10.1186/1471-2288-11-29 PMC3068987

[13] Campbell SH Carey M Sanson-Fisher R , et al. Measuring the unmet supportive care needs of cancer support persons: the development of the support person's unmet needs survey–short form. Eur J Cancer Care (Engl) 2014; 23: 255–262. 24127743 10.1111/ecc.12138

[14] QUIRKOS, https://www.quirkos.com (2023).

[15] CASP, https://casp-uk.net/casp-tools-checklists/systematic-review-checklist/ (2023).

[16] Herzog R Álvarez-Pasquin MJ Díaz C , et al. Are healthcare workers’ intentions to vaccinate related to their knowledge, beliefs and attitudes? A systematic review. BMC Public Health 2013; 13: 154. 23421987 10.1186/1471-2458-13-154 PMC3602084

[17] Fekonja Z Irgolič N Vrbnjak D . Family members’ experiences of everyday caregiving for a family member living with Parkinson’s disease: a qualitative thematic analysis study. BMC Nurs 2024; 23: 98. 38321424 10.1186/s12912-024-01767-6 PMC10845758

[18] Kapelle WM Geerlings AD Mutsaers I , et al. Unveiling the invisible: a qualitative interview study on the impact of young onset Parkinson’s disease on (ex-)partners. J Neurol 2024; 271: 5312–5325. 38861033 10.1007/s00415-024-12474-2 PMC11319367

[19] Krieger T Jozwiak L Ebersbach G , et al. Exploring the lived experiences of individuals with Parkinson’s disease and their relatives: insights into care provision experiences, disease management support, self-management strategies, and future needs in Germany (qualitative study). BMC Neurol 2024; 24: 208. 38890614 10.1186/s12883-024-03696-y PMC11184701

[20] Sharma P Baruah U Yadav A , et al. Understanding psychosocial functioning, caregiver burden, and neuropalliative care in Parkinson’s disease – A mixed-methods study. Ann Indian Acad Neurol 2024; 27: 289–296. 38902870 10.4103/aian.aian_83_24 PMC11232814

[21] White DR Palmieri PA . There is ‘no cure for caregiving’: the experience of women caring for husbands living with Parkinson’s disease. Int J Qual Stud Health Well-Being 2024; 19: 2341989. 38657183 10.1080/17482631.2024.2341989 PMC11044767

[22] Hjelle EG Rønn-Smidt H Haahr A , et al. Filling the gap in service provision. Partners as family carers to people with Parkinson's disease: a Scandinavian perspective. Chronic Illn 2024; 20: 258–270. 37161264 10.1177/17423953231174470

[23] Merritt RK Hotham S Schrag A . Support needs in carers of people with Parkinson’s from early to later stages: a qualitative study with 36 carers in 11 European countries. J Geriatr Psychiatry Neurol 2023; 36: 505–510. 37081815 10.1177/08919887231168404 PMC10578036

[24] Pigott JS Davies N Chesterman E , et al. Delivering optimal care to people with cognitive impairment in Parkinson's disease: a qualitative study of patient, caregiver, and professional perspectives. Parkinsons Dis 2023; 2023: 9732217. 37675146 10.1155/2023/9732217 PMC10480026

[25] Read J Cable S Bartl G , et al. The lived experience of caregiving and perception of service provision among family-caregivers of people with late-stage Parkinson's: a qualitative study. Parkinsons Dis 2023; 2023: 4483517. 36776984 10.1155/2023/4483517 PMC9918353

[26] Read J Frost R Walters K , et al. Transitions and challenges for people with Parkinson's and their family members: a qualitative study. PLoS One 2022; 17: e0268588. 10.1371/journal.pone.0268588 PMC9292070 35849560

[27] Seshadri S Contento A Sugiura K , et al. Parkinson's disease carepartners’ perceptions of the challenges and rewards of caregiving. Am J Hosp Palliat Care 2024; 41: 1442–1450. 38264847 10.1177/10499091231223739 PMC11687559

[28] Geerlings AD Meinders MJ Bloem BR , et al. Using former carers’ expertise in peer support for carers of people with Parkinson's disease. NPJ Parkinsons Dis 2022; 8: 133. 36243820 10.1038/s41531-022-00381-0 PMC9569356

[29] Shahmoon S Limousin P Jahanshahi M . Exploring the caregiver role after deep brain stimulation surgery for Parkinson’s disease: a qualitative analysis. Parkinsons Dis 2023; 2023: 5932865. 37065969 10.1155/2023/5932865 PMC10098415

[30] Bhasin SK Bharadwaj IU . Perceptions and meanings of living with Parkinson’s disease: an account of caregivers lived experiences. Int J Qual Stud Health Well-Being 2021; 16: 1967263. 34414851 10.1080/17482631.2021.1967263 PMC8381973

[31] Jacob SA Wong ZJ Cheong WL , et al. A qualitative exploration of the healthcare challenges and pharmaceutical care needs of people with Parkinson's and their caregivers. Int J Clin Pharm 2022; 44: 53–63. 34318400 10.1007/s11096-021-01312-4 PMC8866252

[32] Zhong X Song PP Wang Z , et al. Resilience building among Chinese family caregivers of older people with Parkinson's disease in Shanghai. Health Soc Care Community 2022; 30: e2395–e2405. 10.1111/hsc.13679 34904318

[33] Deutsch CJ Robertson N Miyasaki JM . Psychological impact of Parkinson disease delusions on spouse caregivers: a qualitative study. Brain Sci 2021; 11: 871. 34210042 10.3390/brainsci11070871 PMC8301855

[34] Rosqvist K Kylberg M Löfqvist C , et al. Perspectives on care for late-stage Parkinson's disease. Parkinsons Dis 2021; 2021: 9475026. 33815742 10.1155/2021/9475026 PMC7987470

[35] Cianfrocca C Caponnetto V Donati D , et al. The opinions and feelings about their educational needs and role of familial caregivers of Parkinson's disease patients: a qualitative study. Acta Biomed 2020; 91: e2020002. 10.23750/abm.v91i12-S.10264 PMC8023112 33263347

[36] Duits A van der Heijden C van Het Hoofd M , et al. Psychosocial needs of patients and spouses justify a position of psychosocial health professionals in the multidisciplinary care for Parkinson's disease. Clin Park Relat Disord 2020; 3: 100064. 34316645 10.1016/j.prdoa.2020.100064 PMC8298809

[37] Jordan SR Kluger B Ayele R , et al. Optimizing future planning in Parkinson disease: suggestions for a comprehensive roadmap from patients and care partners. Ann Palliat Med 2020; 9: S63–S74. 10.21037/apm.2019.09.10 PMC7408313 32036671

[38] McKeown E Saleem T Magee C , et al. The experiences of carers looking after people with Parkinson's disease who exhibit impulsive and compulsive behaviours: an exploratory qualitative study. J Clin Nurs 2020; 29: 4623–4632. 32956513 10.1111/jocn.15499

[39] Armstrong MJ Rastgardani T Gagliardi AR , et al. Barriers and facilitators of communication about off periods in Parkinson's disease: qualitative analysis of patient, carepartner, and physician interviews. PLoS One 2019; 14: e0215384. 10.1371/journal.pone.0215384 PMC6472878 30998707

[40] Nunes SFL Alvarez AM Costa M , et al. Determining factors in the situational transition of family members who care of elderly people with Parkinson’s disease. Texto Contexto Enfermagem 2019; 28: e20170438.

[41] Padovani C Lopes MCL Higahashi IH , et al. Being caregiver of people with Parkinson's disease: experienced situations. Rev Bras Enferm 2018; 71: 2628–2634. 30540037 10.1590/0034-7167-2017-0008

[42] Schwartz R Zulman D Gray C , et al. “It's a disease of families": neurologists’ insights on how to improve communication and quality of life for families of Parkinson's disease patients. Chronic Illn 2020; 16: 201–211. 30208725 10.1177/1742395318799852

[43] Boersma I Jones J Carter J , et al. Parkinson disease patients’ perspectives on palliative care needs: what are they telling us? Neurol Clin Pract 2016; 6: 209–219. 27347438 10.1212/CPJ.0000000000000233 PMC4909525

[44] Habermann B Shin JY . Preferences and concerns for care needs in advanced Parkinson's disease: a qualitative study of couples. J Clin Nurs 2017; 26: 1650–1656. 27571437 10.1111/jocn.13565 PMC5332516

[45] Hurt CS Cleanthous S Newman SP . Further explorations of illness uncertainty: carers’ experiences of Parkinson's disease. Psychol Health 2017; 32: 549–566. 28276746 10.1080/08870446.2017.1283041

[46] Bolland M Guilfoyle A Bucks RS . I’m losing the ‘me’: partners’ experiences of engagement with Parkinson's health professionals. Brain Impair 2015; 16: 116–130.

[47] Martin SC . Psychosocial challenges experienced by partners of people with Parkinson disease. J Neurosci Nurs 2015; 47: 211–222. 26153787 10.1097/JNN.0000000000000141

[48] Hellqvist C Berterö C . Support supplied by Parkinson's disease specialist nurses to Parkinson's disease patients and their spouses. Appl Nurs Res 2015; 28: 86–91. 25908544 10.1016/j.apnr.2014.12.008

[49] Thomas KE Broady TR James CL . Exploring manual handling practices by informal carers. Int J Ther Rehabil 2016; 23: 305–313.

[50] Barken R . Caregivers’ interpretations of time and biography: the experiences of caring for a spouse with Parkinson’s disease. J Contemp Ethnogr 2014; 43: 695–719.

[51] Davis LL Chestnutt D Molloy M , et al. Adapters, strugglers, and case managers: a typology of spouse caregivers. Qual Health Res 2014; 24: 1492–1500. 25189535 10.1177/1049732314548879 PMC4350575

[52] Leiknes I Høye S . Family caregivers’ experiences of provided home care to persons with Parkinson's disease. Nordisk Sygeplejeforskning 2012; 2: 29–44.

[53] van Rumund A Weerkamp N Tissingh G , et al. Perspectives on Parkinson disease care in Dutch nursing homes. J Am Med Dir Assoc 2014; 15: 732–737. 24984788 10.1016/j.jamda.2014.05.009

[54] Beaudet L Ducharme F . Living with moderate-stage Parkinson disease: intervention needs and preferences of elderly couples. J Neurosci Nurs 2013; 45: 88–95. 23422694 10.1097/JNN.0b013e3182828ff4

[55] Abendroth M Lutz BJ Young ME . Family caregivers’ decision process to institutionalize persons with Parkinson's disease: a grounded theory study. Int J Nurs Stud 2012; 49: 445–454. 22036578 10.1016/j.ijnurstu.2011.10.003

[56] Tan SB Williams AF Morris ME . Experiences of caregivers of people with Parkinson's disease in Singapore: a qualitative analysis. J Clin Nurs 2012; 21: 2235–2246. 22788558 10.1111/j.1365-2702.2012.04146.x

[57] Duncan GF Rositano P . Parkinson's disease in regional Australia. Rural Remote Health 2011; 11: 1658. 22032468

[58] Hounsgaard L Pedersen B Wagner L . The daily living for informal caregivers with a partner with Parkinson’s disease – An interview study of women’s experiences of care decisions and self-management. J Nurs Healthc Chronic Illn 2011; 3: 504–512.

[59] McLaughlin D Hasson F Kernohan WG , et al. Living and coping with Parkinson's disease: perceptions of informal carers. Palliat Med 2011; 25: 177–182. 20952448 10.1177/0269216310385604

[60] van der Eijk M Faber MJ Al Shamma S , et al. Moving towards patient-centered healthcare for patients with Parkinson's disease. Parkinsonism Relat Disord 2011; 17: 360–364. 21396874 10.1016/j.parkreldis.2011.02.012

[61] Hasson F Kernohan WG McLaughlin M , et al. An exploration into the palliative and end-of-life experiences of carers of people with Parkinson's disease. Palliat Med 2010; 24: 731–736. 20525749 10.1177/0269216310371414

[62] Giles S Miyasaki J . Palliative stage Parkinson's disease: patient and family experiences of health-care services. Palliat Med 2009; 23: 120–125. 19098110 10.1177/0269216308100773

[63] McCabe MP Roberts C Firth L . Work and recreational changes among people with neurological illness and their caregivers. Disabil Rehabil 2008; 30: 600–610. 17852325 10.1080/09638280701400276

[64] Hudson PL Toye C Kristjanson LJ . Would people with Parkinson's disease benefit from palliative care? Palliat Med 2006; 20: 87–94. 16613404 10.1191/0269216306pm1108oa

[65] Habermann B Davis LL . Caring for family with Alzheimer's disease and Parkinson's disease: needs, challenges and satisfaction. J Gerontol Nurs 2005; 31: 49–54. 10.3928/0098-9134-20050601-11 16138530

[66] Davey C Wiles R Ashburn A , et al. Falling in Parkinson's disease: the impact on informal caregivers. Disabil Rehabil 2004; 26: 1360–1366. 15742981 10.1080/09638280400000195

[67] Birgersson AM Edberg AK . Being in the light or in the shade: persons with Parkinson's disease and their partners’ experience of support. Int J Nurs Stud 2004; 41: 621–630. 15240086 10.1016/j.ijnurstu.2004.01.007

[68] Perrin PB Henry RS Donovan EK , et al. Parkinson’s family needs and caregiver mental health: a cross-cultural comparison between Mexico and the United States. Neurorehabilitation 2019; 45: 433–442. 31868689 10.3233/NRE-192894 PMC7025758

[69] Serio C Kreutzer J Witol A . Family needs after traumatic brain injury: a factor analytic study of the Family Needs Questionnaire. Brain Inj 1997; 11: 1–10. 9012547 10.1080/026990597123764

[70] Kristjanson LJ Aoun SM Oldham L . Palliative care and support for people with neurodegenerative conditions and their carers. Int J Palliat Nurs 2006; 12: 368–377. 17077795 10.12968/ijpn.2006.12.8.368

[71] Schrag A Morley D Quinn N , et al. Impact of Parkinson's disease on patients’ adolescent and adult children. Parkinsonism Relat Disord 2004; 10: 391–397. 15465394 10.1016/j.parkreldis.2004.03.011

[72] Zauszniewski JA Sweetko JS She HY , et al. Documenting the need for teaching resourcefulness skills to family caregivers. Appl Nurs Res 2022; 67: 151627. 36116865 10.1016/j.apnr.2022.151627

[73] Shin JY Habermann B . Key activities of caregivers for individuals with Parkinson disease: a secondary analysis. J Neurosci Nurs 2020; 52: 284–288. 33156150 10.1097/JNN.0000000000000544

[74] Sturm D Folkerts AK Kalbe E . Easing burden and stress: intervention needs of family members of patients with Parkinson's disease. J Parkinsons Dis 2019; 9: 221–227. 30584152 10.3233/JPD-181456

[75] Baik JS Kim JS Koh SB , et al. Patients and their caregivers’ burdens for Parkinson's disease in Korea. J Mov Disord 2017; 10: 109–115. 28950688 10.14802/jmd.17053 PMC5615179

[76] Olsson Y Clarén L Alvariza A , et al. Health and social service access among family caregivers of people with Parkinson's disease. J Parkinsons Dis 2016; 6: 581–587. 27176624 10.3233/JPD-160811

[77] Lageman SK Mickens MN Cash TV . Caregiver-identified needs and barriers to care in Parkinson's disease. Geriatr Nurs 2015; 36: 197–201. 25744557 10.1016/j.gerinurse.2015.01.002

[78] Parrish M Giunta N Adams S . Parkinson's disease caregiving: implications for care management. Care Manag J 2003; 4: 53–60. 14502879 10.1891/cmaj.4.1.53.57471

[79] Fidder H Jaski JJ Elbertse E , et al. Parkinson rehabilitation in nursing homes: a qualitative exploration of the experiences of patients and caregivers. Eur Geriatr Med 2022; 13: 1197–1210. 35543902 10.1007/s41999-022-00647-z PMC9092320

[80] Lawson RA Yarnall AJ Johnston F , et al. Cognitive impairment in Parkinson's disease: impact on quality of life of carers. Int J Geriatr Psychiatry 2017; 32: 1362–1370. 27925292 10.1002/gps.4623 PMC5724657

[81] Prado L Hadley R Rose D . Taking time: a mixed methods study of Parkinson's disease caregiver participation in activities in relation to their wellbeing. Parkinsons Dis 2020; 2020: 7370810. 32351682 10.1155/2020/7370810 PMC7171685

[82] A'Campo LE Wekking EM Spliethoff-Kamminga NG , et al. The benefits of a standardized patient education program for patients with Parkinson's disease and their caregivers. Parkinsonism Relat Disord 2010; 16: 89–95. 19674927 10.1016/j.parkreldis.2009.07.009

[83] A'Campo LE Spliethoff-Kamminga NG Roos RA . An evaluation of the patient education programme for Parkinson's disease in clinical practice. Int J Clin Pract 2011; 65: 1173–1179. 21951713 10.1111/j.1742-1241.2011.02765.x

[84] A'Campo LE Spliethoff-Kamminga NG Macht M , et al. Caregiver education in Parkinson's disease: formative evaluation of a standardized program in seven European countries. Qual Life Res 2010; 19: 55–64. 19946755 10.1007/s11136-009-9559-y PMC2804793

[85] Kinchin I Edwards L Adrion E , et al. Care partner needs of people with neurodegenerative disorders: what are the needs, and how well do the current assessment tools capture these needs? A systematic meta-review. Int J Geriatr Psychiatry 2022; 37: 10.1002/gps.5764. 10.1002/gps.5764 PMC9328373 35665539

[86] Dahodwala N Shah K He Y , et al. Sex disparities in access to caregiving in Parkinson disease. Neurology 2018; 90: e48–e54. 10.1212/WNL.0000000000004764 PMC10681055 29196580

[87] Rosqvist K Schrag A Odin P , et al. Caregiver burden and quality of life in late stage Parkinson's disease. Brain Sci 2022; 12: 111. 35053854 10.3390/brainsci12010111 PMC8773513

[88] Lee GB Woo H Lee SY , et al. The burden of care and the understanding of disease in Parkinson's disease. PLoS One 2019; 14: e0217581. 10.1371/journal.pone.0217581 PMC6544353 31150470

[89] Shin H Lee JY Youn J , et al. Factors contributing to spousal and offspring caregiver burden in Parkinson's disease. Eur Neurol 2012; 67: 292–296. 22517329 10.1159/000335577

[90] Boumans NPG Dorant E . The relationships of job and family demands and job and family resources with family caregivers’ strain. Scand J Caring Sci 2021; 35: 567–576. 32400014 10.1111/scs.12873 PMC8247051

[91] Gülke E Pötter-Nerger M . Caregiver burden in partners of Parkinsonian patients with deep brain stimulation. Brain Sci 2022; 12: 238. 35204001 10.3390/brainsci12020238 PMC8870343

[92] Crespo-Burillo JA Rivero-Celada D Saenz-de Cabezón A , et al. Deep brain stimulation for patients with Parkinson's disease: effect on caregiver burden. Neurologia (Engl Ed) 2018; 33: 154–159. 27443241 10.1016/j.nrl.2016.05.017

[93] Theed R Eccles F Simpson J . Experiences of caring for a family member with Parkinson's disease: a meta-synthesis. Aging Ment Health 2017; 21: 1007–1016. 27802771 10.1080/13607863.2016.1247414

[94] Chen Y Zhou W Hou L , et al. The subjective experience of family caregivers of people living with Parkinson's disease: a meta-ethnography of qualitative literature. Aging Clin Exp Res 2022; 34: 959–970. 34648175 10.1007/s40520-021-01995-9

