# Supplementary material for: Care partner needs in Parkinson's disease: A systematic review of qualitative and quantitative data
Source: J Parkinsons Dis. 2025 May 30:1877718X251344066. Online ahead of print. doi: 10.1177/1877718X251344066 (PMC13347510; doi:10.1177/1877718X251344066)
Supplement: Supplementary file 1 [file sj-docx-1-pkn-10.1177_1877718X251344066.docx]

**Supplemental Material**

**Care partner needs in Parkinson’s disease: A systematic review of qualitative and quantitative data**

**Theme definitions:**

**1. The need for information:**

Providing facts on any topic of relevance to the care partner that would assist in their role as a care partner or otherwise help them.

**2. The need to be heard:**

The desire of the care partner to have their concerns acknowledged and heard by others (person with PD, healthcare practitioners, family, other care partners etc.), to be recognized as a care partner.

**3. PD healthcare:**

Structural shortcomings of the healthcare system surrounding the person with PD and the care partner with direct effect on the care partner and expressed by the care partner.

**4. Emotional support:**

Receiving empathy, compassion and other ways of emotional support from either their social networks or healthcare practitioners that contributes to the well-being of the care partner.

**5. Daily living (assisted living and home adaptation):**

Matters regarding home adjustments, professional care at home, and assisted living facilities.

**6. Financial support:**

Statements regarding the need for financial support or financial strain related directly or indirectly to PD care.

**7. Skills for providing care:**

Providing instruction on any skills of relevance to the care partner that would assist in their role as a care partner.

**8. Care partner physical well-being:**

Matters related to the physical health of the care partner that results from or affects their role as a care partner.

**9. Respite care:**

The temporary relief from their role/duties as a care partner and potential facilitators of this relief.
